# Climate change and mental health: risks, impacts and priority actions

**DOI:** 10.1186/s13033-018-0210-6

**Published:** 2018-06-01

**Authors:** Katie Hayes, G. Blashki, J. Wiseman, S. Burke, L. Reifels

**Affiliations:** 1 Dalla Lana School of Public Health, University of Toronto, Toronto, ON Canada; 2 Nossal Institute for Global Health, University of Melbourne, Carlton, VIC Australia; 3 Melbourne Sustainable Society Institute, University of Melbourne, Carlton, VIC Australia; 4 Australian Psychological Society, Level 11, 257 Collins St, Melbourne, VIC Australia; 5 Centre for Mental Health, Melbourne School of Population and Global Health, The University of Melbourne, Carlton, VIC Australia

**Keywords:** Climate change, Mental health, Attribution, Mitigation, Adaptation

## Abstract

**Background:**

This article provides an overview of the current and projected climate change risks and impacts to mental health and provides recommendations for priority actions to address the mental health consequences of climate change.

**Discussion and conclusion:**

The authors argue the following three points: firstly, while attribution of mental health outcomes to specific climate change risks remains challenging, there are a number of opportunities available to advance the field of mental health and climate change with more empirical research in this domain; secondly, the risks and impacts of climate change on mental health are already rapidly accelerating, resulting in a number of direct, indirect, and overarching effects that disproportionally affect those who are most marginalized; and, thirdly, interventions to address climate change and mental health need to be coordinated and rooted in active hope in order to tackle the problem in a holistic manner. This discussion paper concludes with recommendations for priority actions to address the mental health consequences of climate change.

## Background

It is well understood that human health is threatened by the impacts of climate change [1–3]. In the 2017 Lancet Countdown on Climate Change and Health, authors state: “The human symptoms of climate change are unequivocal and potentially irreversible—affecting the health of populations around the world today” [4]. Climate change is no longer a looming threat but rather a destructive reality with dire predictions for the future. The World Health Organization (WHO) estimates an increase of 250,000 excess deaths per year between 2030 and 2050 due to the “well understood impacts of climate change” [5]. Impacts include heat-related morbidity and mortality, increases in vector-borne diseases (e.g. dengue fever, malaria), increased respiratory illness, and morbidity and mortality due to extreme weather events [6, 7]. The lesser-known, and often overlooked, effects of climate change include the risks and impacts to mental health—the focus of this article.

Mental health refers not just to mental illness, mental problems, and mental disorders, but also includes states of mental wellness, emotional resilience and psychosocial wellbeing [8–11]. Psychosocial wellbeing is the interplay between social and psychological conditions that shape human welfare; a broad term which encompasses the states of being mentally healthy, experiencing mental problems, and mental illness [7, 10].

Investigating the current state of evidence and knowledge about the climate change impacts to mental health, this article pays particular attention to the inequitable impacts of climate change on the mental health of marginalized and vulnerable populations. We argue the following three points: firstly, while attribution of mental health outcomes to specific climate change risks remains challenging, there are a number of opportunities available to advance the field of mental health and climate change with more empirical research in this domain; secondly, the risks and impacts of climate change on mental health are already rapidly accelerating, resulting in a number of direct, indirect, and overarching effects that disproportionally affect those who are most marginalized; and finally, interventions to address climate change and mental health need to be coordinated and rooted in active hope in order to tackle the problem in a holistic manner. This paper explores each of these facets and concludes with recommendations to enhance the state of knowledge and actions on climate change and mental health. Before diving into the topic area, a brief overview of climate change and health effects are noted in the sections below.

### Climate change and health

An extensive body of research continues to strengthen knowledge about the impact of climate change on physical health, including for example, a rise in vector-borne, water and food-borne diseases; an increase in acute and chronic respiratory conditions (including asthma and allergies); and, heat-related and extreme weather-related morbidity and mortality [2–4, 12]. Indirect health implications that are increasingly recognised in global reports on climate change and health include illness related to food and water safety, under-nutrition related to food insecurity, malignant melanoma from UV exposure, and chronic kidney disease from dehydration [4]. In late 2017, the Lancet released its first, full tracking report on climate change and health. In this report, there is an explicit request for more information on, and actions to address, the “often-unseen” impacts of climate change on human health, notably, the mental health consequences of climate change [4].

### Climate change and mental health

The expanding research literature on climate change and mental health includes increasing evidence that extreme weather events—which are more frequent, intense, and complex under a changing climate—can trigger post-traumatic stress disorder (PTSD), major depressive disorder (MDD), anxiety, depression, complicated grief, survivor guilt, vicarious trauma, recovery fatigue, substance abuse, and suicidal ideation [13–26]. Incremental climate changes, such as rising temperatures, rising sea levels, and episodic drought, can change natural landscapes, disrupt food and water resources, change agricultural conditions, change land use and habitation, weaken infrastructure and give rise to financial and relationship stress, increase risks of violence and aggression, and displacement of entire communities [4, 18, 23, 27, 28]. The overarching threats of a changing climate, can also incite despair and hopelessness as actions to address the ‘wicked problem’ of climate change seem intangible or insignificant in comparison to the scale and magnitude of the threats [29]. Paradoxically, these same disastrous circumstances may also inspire altruism, compassion, optimism, and foster a sense of meaning and personal growth (otherwise referred to as post-traumatic growth) as people band together to salvage, rebuild, and console amongst the chaos and loss of a changing climate [30, 31].

### Climate change and health inequity

It is well understood that climate change augments existing inequalities, rendering those most marginalized at greater peril to the health consequences of a changing climate [4, 32, 33]. In fact, the first key message from the Lancet’s Countdown on Climate Change and Health report emphasizes the disproportionate impact climate change has on the world’s most marginalized people and the consequential impacts this has on these populations if social and environment justice concerns are not addressed [4]. Watts et al. state: “By undermining the social and environmental determinants that underpin good health, climate change exacerbates social, economic, and demographic inequalities, with the impacts eventually felt by all populations” [4]. Those who are at greatest risk to the effects of climate change are those who are most marginalized based on socially and environmentally mediated factors, such as socioeconomic status, culture, gender, race, employment, and education [15, 34]. Marginalized groups who tend to be the most affected by the mental and physical health implications of climate change are: Indigenous peoples, children, seniors, women, people with low-socioeconomic status, outdoor labourers, racialized people, immigrants, and people with pre-existing health conditions [2, 3, 7, 13, 22, 23, 33, 35, 36]. Importantly, these marginalized groups are not homogenous. People may experience intersections of marginalization based on a variety of the above social indicators.

### Exploring the relationship between mental health and climate change

An updated overview of recent evidence on the mental health implications of climate change is timely given the ongoing, rapid expansion of research in the broad field of health and climate change as well as increasing public concern about climate change trends and risks.^1^ Since 2007, media reports on climate change and health have increased by 78% and the academic literature on climate and health issues has tripled [4]. There is also increasing public and academic recognition of the extent to which rising global temperatures threaten planetary and human health [38, 39]. While public awareness about the health implications of climate change continues to grow, the topic of mental health is frequently absent from this discourse. In some ways, this reflects the global discourse, where, in comparison to physical health, mental health *in general* has been neglected.

Globally, the prevalence of mental health issues is extremely high even without considering the added mental health consequences of a changing climate. Based on a 10-year systematic analysis of global burden of disease from 1990 to 2010, Murray et al. find that mental illness comprises 7.8% of the global burden of disease [40]. Mental and behavioural disorders also account for the greatest global burden of years lived with a disability (YLDs) [41]. Vigo et al. contend that these figures are actually much higher if co-morbidities related to mental illness are considered within burden of illness studies and if a more accurate definition of mental illness is used, a definition that includes health behaviours like self-harm and suicide [42]. The failure of global investment in mental health care to address the consequences and impacts of rapidly escalating levels of mental illness has been described as a “global tragedy” reflecting a long “legacy of the neglect and marginalization of mental health” [41]. Similarly, authors from the 2016 Lancet report on sustainable development and global mental health describe the state of mental health as the “most neglected of all human health conditions” and a “failure of humanity [43]. The inattention to mental health is of particular concern in the field of climate change and mental health given the evidence that psychological impacts from any form of disaster exceed physical injury by 40–1 [44], and that since 2000 the frequency of climate change-related weather disasters has increased by 46% [4]. Crucially, it is the most marginalized who are especially vulnerable to climate change’s impacts on mental health. As McMichael notes, climate change acts as a health “threat amplifier”, compounding existing social injustices [32]. There is, therefore, a strong case for continuing to explore and communicate research and policy learning about the relationship between climate change and mental health—especially as the topic area pertains to health equity.

## Discussion

### Part 1. Exploring the challenges and opportunities of attribution

The lack of attention to the topic of climate change and mental health is often imputed to the challenges of attribution. Attribution in this case is the scientific association between greenhouse gas emissions and meteorological change on the one hand, and between climate change-related meteorological change and mental health effects on the other. There is now an increasingly strong body of literature which highlights the causal linkages between climate change and extreme weather events (see [45–50]). One of the key messages within this literature is that while we cannot say with certainty that any one specific extreme weather event is directly caused by climate change; we do know that because of climate change, extreme weather is more generally on the rise, making extreme weather events more frequent, intense, and complex. In other words, climate change therefore ‘loads the dice’ for more weather extremes.

Within the disaster mental health literature, the links between extreme weather events and mental health effects are well established (see for example [51–59]). However, many of these studies tend not to connect extreme weather to a changing climate—instead referring to extreme weather events as natural disasters rather than events linked to anthropogenic climate change. Studies within this domain tend to focus on mental health outcomes of specific hazard events (e.g. the 2004 Tsunami in Malaysia, Hurricane Katrina in 2005; Southern Alberta floods in 2013) positioning each hazard as an isolated incident unconnected to the wicked problem of climate change. The risk of overlooking or minimizing the role of climate change within these hazardous events is that this creates a reactive culture of emergency response that inhibits appropriate and effective adaptation planning and preparation for complex emergencies that a changing climate can create.

An additional concern is that much of the disaster mental health research has traditionally focussed on three distinct phases (the emergency and crisis stage, the post-impact stage, and the rehabilitation and recovery phase) [56], while little attention has been paid to psychosocial phenomena that can occur during the pre-disaster phase. Such phenomena include, for example, heightened anxiety levels, feelings of impending doom, hopelessness, and fatalism that can be triggered by approaching extreme events or associated weather warnings; and which may also be amplified due to the perceived risk of subacute, environmental changes like rising temperatures and episodic droughts [13, 20, 22].

#### Key challenges of attributing climate change to mental health

Attribution related to climate change and mental health can be challenging for four key reasons: firstly, there is a risk of pathologising common transitory distress responses to abnormal events and underdiagnosing mental health effects of a changing climate; secondly, there is a wide array of potential climate change and mental health outcomes related to a changing climate; thirdly, there is substantial scope with respect to the timing of the climate change effects on mental health, thus causal links become harder to determine; and finally, attribution related to climate change and mental health is not well understood because of the complex interaction between mental health and other social determinants of health.

There is a simultaneous risk of pathologising ‘normal’ responses to a changing climate and of underdiagnosing the real mental health effects of a changing climate. Pathologising ‘normal’ responses to disaster situations may result in a failure to differentiate between mild transitional distress or grief and more severe, persistent mental health problems. Both overinflating or underinflating mental health outcomes associated with climate change can lead to erroneous prevalence estimates and skewed assumptions about mental health service needs. A further consideration noted by Whaley in the aftermath of Hurricane Katrina is that in some cases medical professionals did not assess pre-existing mental health conditions and, therefore, attributed disaster trauma as a typical stress response, or alternatively diagnosed patients with stress response when in fact there were much larger mental health issues related to the effects of the Hurricane that went undiagnosed [60]. Crucially important to consider is that pre-existing mental health conditions or problems can be exacerbated or even triggered by changes in climate [9, 61].

As noted earlier, mental health includes states of mental wellness as well as mental problems and disorders. With this in mind, the current application of tools to assess mental health have some limitations. Researchers tend to conceptualize mental health solely as mental illness and mental problems, administering surveys using validated instruments that assess mental health problems and issues like: generalized anxiety disorder (using the general anxiety disorder, GAD-2 questionnaire), PTSD (PCL-6 checklist), and psychological distress (via the general health questionnaire, GHQ-12) following an extreme weather event [52, 53, 62–66]. Few empirical studies that use these survey methods capture positive psychological consequences of extreme weather events, like feelings of compassion, altruism, sense of meaning, post-traumatic growth, or even increased acceptance of climate change and engagement with climate mitigation. This information can elucidate the complexity of mental health impacts from a changing climate and also help to understand any predisposing factors that may influence positive mental health outcomes and build psychosocial resilience.

Timing of psychosocial implications from climate related hazards poses another challenge. Scholars have discovered wide-ranging timeframes for the psychosocial impacts to manifest. Azuma et al. find that the incidence of psychological disorders (including PTSD) tended to be most significant within 6 months after a flood [62]. Kessler et al. conducted interviews with Hurricane Katrina survivors 5–8 months post-event and 1-year post-event, these authors found an increase in mental health disorders as time progressed [65]. For example, PTSD increased from 14.9% at 5–8 months to 20.9% after 1 year. Anderson et al. suggest that psychosocial impacts tend to peak within the first-year, post-extreme weather event [67]. Tunstall et al. on the other hand, found that residents who experience significant flooding self-report long-term psychosocial impacts (namely anxiety when it rains) from 2.5 to 5 years post-flooding [51]. A recent news article suggests that over 7000 people who experienced Hurricane Katrina in 2005 are still receiving mental health care for trauma associated with the Hurricane [68]. Noting that psychosocial health outcomes can have latent effects, or that these outcomes can occur as a result of sequelae, knowing how and when to study the climate change-related impacts and psychosocial outcomes becomes increasingly challenging—especially if the aim is to demonstrate the magnitude and attribution of effects.

Distilling the precise impact of climate change on mental health can be difficult to separate from other social determinants. As Watts et al. note, measuring the impacts of climate change on mental health is challenging not only because of attribution but also because of the “complicated nature of mental health, which embraces a diverse array of outcomes (e.g. anxiety and mood disorders), many of which co-occur and all of which vary with contexts and during lifetimes. Mental health impacts are often products of long and complex causal pathways, many of which can be traced back to distal but potent root causes, such as famine, war, and poverty, of which climate change is an accelerator” [4]. Mental health, like physical health, is shaped by social and ecological factors that can influence—and often amplify—other determinants of health, like a changing climate.

#### Opportunities of attributing mental health to climate change

It is important to locate climate change within the discourse on mental health because the frequency, intensity, duration, and complexity of climate change effects is on the rise and thus climate-related mental health outcomes are also increasing—adding to the already burgeoning burden of mental illness and mental problems globally. Acknowledging the mental health consequences of climate change helps the mental health community to discern and anticipate patterns of mental illness, like for example PTSD following extreme weather events. Also, an understanding of the unequal impacts of climate change on marginalized groups supports public health prevention strategies that seek to protect those most susceptible to mental illness and mental problems.

There is a risk that climate-related psychosocial consequences may become diluted in the high prevalence of mental health disorders globally; therefore, there is a need for additional research within the specific domain of climate change and mental health. If there is a better understanding of the linkages between climate change and mental health, there are more opportunities to understand and address climate change and mental health via actions rooted in climate change mitigation and adaptation that support psychosocial resilience. Specifically, the field requires more empirical research on the mental health consequences of climate change, especially as this research relates to marginalized communities and the risks and impacts associated with chronic climate change-related hazards and consequences (like sea-level rise, rising temperatures, and ecological degradation). To address the overarching interplay of social and environmental determinants of health that can magnify climate change-related risks on mental health, a health equity approach to this area of study is required. Secondly, there is a need to better understand climate change-related hazards within the context of mental health sequelae. Research in this area could help to explore the complexity of climate change and mental health attribution by recognizing the role of predisposing mental health conditions while also taking into account the perceived and actual risks and impacts related to a changing climate. This type of research can support a better understanding of the triggers and timing of climate change-related mental health effects as well as support policy and program development for mental health resources.

Attributing mental health outcomes to climate change also presents opportunities to assess, build, and strengthen mental health systems. In 2015, the World Health Organization (WHO) set forth the *framework for building climate resilient health systems* [69]. This framework provides guidance for health professionals to predict, prevent, and prepare for climate change-related shocks with the ultimate aim of protecting population-level health by increasing health systems’ capacity to cope, adapt, sustain, and strengthen in the wake of a changing climate. While the framework overlooks the intricacies of mental health systems—like the current and prevailing lack of mental health infrastructure, funding, and resourcing globally—it does provide the necessary guidance to build mental health systems resiliency. Articulating climate change as a determinant of mental health not only brings awareness of the broad consequences of climate change on health but also supports the enhancement of mental health systems.

In sum, the key benefits of understanding the linkages between climate change and mental health include: enhanced knowledge of patterns of illness; an added emphasis on the global call to action to reduce and address climate change risks and impacts; in-depth knowledge of the risks and impacts to marginalized communities; and, better planning for mental health response and mental health systems resiliency.

### Part 2. Current risks and impacts of climate change on mental health

It is challenging for people to recognise changes in climate because these changes appear distant or abstract—especially because climate is often confused or lost in perceptions about weather or seasonal change [23]. The influential sociologist Anthony Giddens refers to this space and time distancing of the climate change problem as the Giddens Paradox [70]. The Giddens Paradox states that: “since the dangers posed by global warming aren’t tangible, immediate or visible in the course of day-to-day life, many will sit on their hands and do nothing of a concrete nature about them. Yet waiting until such dangers become visible and acute—in the shape of catastrophes that are irrefutably the result of climate change—before being stirred to serious action will be too late” (p. 2). Marshall contends that part of the time and space distancing of the climate change problem, and thus the reluctance to act, is reinforced by the Western political discourse on climate change as a future-facing problem that intentionally overlooks the centuries of industrialization, fossil fuel consumption, and land degradation that contribute to anthropogenic climate change [71]. Marshall calls for a reckoning with this discourse by noting:

“Climate change is a future problem. But it is also a past problem and a present problem. It is better thought of as a developing process of long-term deterioration, called, by some psychologists, a “creeping problem.” The lack of a definite beginning, end, or deadline requires that we create our own timeline. Not surprisingly, we do so in ways that remove the compulsion to act. We allow just enough history to make it seem familiar but not enough to create a responsibility for our past emissions. We make it just current enough to accept that we need to do something about it but put it just too far in the future to require immediate action” [71].

Noting the Giddens Paradox and the reckoning that Marshall asks us to have with the “creeping problem” of climate change, it becomes important to confront the current mental health consequences related to climate change that are happening now [70, 71]. To do so, it is useful to explore the conceptual framework of climate change and mental health developed by Berry et al. [15]. These authors organize climate change-related hazards into three categories: acute (flooding, hurricanes, etc.), sub-acute (pervasive drought), and chronic (rising sea-level, increasing temperatures). These climate change-related hazards lead to a variety of direct, indirect, and overarching psychosocial consequences that are occurring now—disproportionately affecting those most marginalized.

Direct psychosocial consequences of climate change include trauma related to extreme weather events, like floods, hurricanes, wildfires, and heat waves [15, 37]. Indirect mental health consequences of climate change occur through social, economic, and environmental disruptions (e.g. famine, civil conflict, displacement, and migration) related to a changing climate [15, 37]. The overarching psychosocial consequences of climate change relate to the long-term emotional distress caused by awareness of the threats and impacts of climate change on the current and future wellbeing of the earth and its inhabitants. The multidimensional climate change and mental health pathway leads to a variety of unequal psychosocial consequences explored below.

#### Direct mental health consequences of climate change

There is now an extensive and rapidly expanding body of research exploring the current mental health consequences of climate change-related extreme weather events. Extreme heat events and humidity have been noted to increase hospital admissions for mood and behavioural disorders, including schizophrenia, mania, and neurotic disorders [72, 73]. Scholars in the field note that heat-related mental health morbidity tends to occur most often in people with impaired thermoregulation, namely people with pre-existing mental health illness and problems, people taking prescription medications (specifically lithium, neuroleptic and anticholinergic drugs), and those with substance abuse (alcohol and drugs) problems [35, 36, 74]. Extreme heat is also linked with an increased risk of wildfires, which also directly impact mental health. Bryant et al. mapped the psychological outcomes of the Black Saturday bushfires in Victoria, Australia; in communities most at risk to the impacts of bushfires, these authors found incidences of PTSD, psychological distress, and depression related to the fires [75].

The direct mental health consequences related to flooding and hurricanes are also well documented (see [51–55, 60, 62, 76–81]). In a study of 30 locations in England and Wales, Tunstall et al. conducted interview surveys with residents affected by flooding. They found that psychological impacts were more commonly reported than physical effects [51]. One study researching the psychosocial impacts following Hurricane Katrina estimates that 20–35% of survivors experienced some form of mental health issue following the disaster [60]. Galea et al. reported a 31.2% prevalence of anxiety-mood disorders amongst Hurricane Katrina survivors [81], while Rhodes and Chan found that nearly half (47.7%) of marginalised community members of New Orleans (mainly low-income, African American women) showed probable signs of PTSD after Hurricane Katrina [82].

While PTSD is often reported as one of the most severe mental health impacts related to acute climate change-related disasters, there have also been increasing reports of suicide and suicidal ideation following extreme weather events. Chand et al. note one Italian study that found higher rates of suicide in northern communities with greater climate variability [72, 83]. Dodgen et al. highlight the risk of homicide-suicides after extreme weather events by noting the doubling of these incidents following Hurricane Andrew in 1992 in Miami-Dade County [74]. There is also observed evidence of increased suicidal thoughts (from 2.8 to 6.4%) and plans to commit suicide (from 1.0 to 2.5%) 18-months after an extreme weather event [74]. Notably, however, the overall evidence linking changing climate and suicide is still inconclusive. Studies on suicidality in natural disaster contexts, for example, vary considerably in study methodology and timeframes considered, with recent reviews indicating divergent trends in suicidality rates following exposure to extreme events, ranging from an initial decline, to neutral effects, all the way to a delayed increase in suicidality [84].

On a deeper level, the psychological responses of communities and individuals to disasters are complex and varied and do not necessarily simply result in more mental illnesses. Rebecca Solnit, in *A Paradise Built in Hell*, usefully describes the complicated psychosocial consequences that can arise after an extreme weather event as, “that sense of immersion in the moment and solidarity with others caused by the rupture in everyday life, an emotion graver than happiness but deeply positive. We don’t even have a language for this emotion, in which the wonderful comes wrapped in the terrible, joy in sorrow, courage in fear. We cannot welcome disaster, but we can value the responses, both practical and psychological” [85]. Exploring the complexity of psychological responses in the book, *Climate change and human well*-*being*, Weissbecker et al., discuss the full spectrum of psychosocial consequences of climate change-related events ranging from mental illness to more positive experiences like ‘Post Traumatic Growth’ (PTG), empathy, compassion, altruism, and emotional resilience [25].

#### Indirect mental health consequences of climate change

The indirect mental health consequences of climate change can occur as a result of damages to physical and social infrastructure, physical health effects, food and water shortages, conflict, and displacement from acute, subacute, and chronic climactic changes [15]. One of the most well-documented climate hazards that indirectly influences mental health is drought. Long-term droughts affect food and water supplies and can subsequently affect the economic and mental wellbeing of land-based workers, most often impacting those living in rural and remote communities [58, 86, 87]. In a quantitative analysis of drought and distress in Australia over a 7-year period, authors found that rural dwellers experience more distress due to the droughts than their urban counterparts [86]. In a systematic review of the literature, authors note the most prominent causal pathway linking drought and mental health is via the economic effects from land degradation [58]. These effects are most prominent amongst farmers whose economic livelihoods depend on environmental conditions. Exemplifying this, a 2008 study in New South Wales, Australia reports that nearly three quarters of farmers report stress related to persistent drought [88]. Some authors also suggest that income insecurity related to drought increases the risk for suicide among farmers [9, 89].

Long-term drought has also been increasingly linked to conflict and forced migration, which can influence psychosocial outcomes like the propensity for stress, PTSD, anxiety, and trauma [90]. The Institute for Environment and Human Security of the United Nations University estimates that migration due to climate change may vary drastically, citing estimates of between 25 million to 1 billion by 2050, with 200 million as the most frequently cited estimate [91]. The rise in the number of ‘climate migrants’ has been identified as a significant risk by an increasing number of defence and security experts [92, 93]. Gleick postulates that the civil conflict in Syria can be traced to the agricultural failures in 2006–2009 and the returning drought in 2011 [90]. In 2011, over 1.5 million Syrians moved from rural, agricultural areas to urban areas seeking refuge from the pervasive drought, failed agriculture, and lack of food and water [90]. Pervasive ecological degradation, poor policy response to water and food insecurity, and ongoing tensions between rural and urban community members, have arguably all contributed to civil unrest and ongoing conflict in Syria [90]. According to the United Nations, the number of displaced Syrians has reached over 5 million people in the past 5 years [94]. Migration from a war-torn country to a host country where culture, language, and lifestyle may be vastly different may also contribute to psychosocial malaise as displaced migrants can face stressors associated with xenophobia and racism from people in their new host country [90]. Conversely as Siriwardhana and Stewart note, displacement may also support psychosocial resilience by fostering hope and belonging for refugees in host countries where they feel welcomed, safe, and experience better living conditions [95].

At the community level the indirect mental health consequences of climate change are understudied. These consequences may include things like a diminishment in community cohesion, the loss of community identity, threats to a sense of continuity and sense of belonging as people are forced to move in and out of communities because of environmental stressors, and an undermining of cultural integrity if people have to leave their homelands [23]. Migration challenges the identity, sovereignty and heritage of people who have to leave their homelands. It also challenges the integrity and continuity of people’s traditional ways of life. Threats to community health also include an increased likelihood of criminal behaviour, violence and aggression as community members experience various stressors related to climate change [23].

#### Overarching psychosocial consequences of global climate change

Awareness of the looming threats and current risks and impacts of climate change presents challenges to emotional and social wellbeing [37]. Since early 2007, environmental philosopher Glenn Albrecht and colleagues have taken note of emotional distress related to the awareness of the overarching problem humans face as a result of global climate change [96]. Albrecht et al. suggest that this awareness contributes to ‘psychoterratic syndromes’. Psychoterratic syndromes include phenomena such as ‘ecoanxiety’, ‘ecoparalysis’, and ‘solastalgia’. ‘Ecoanxiety’ refers to the anxiety people face from constantly being surrounded by the wicked and threatening problems associated with a changing climate [96]. ‘Ecoparalysis’ refers to the complex feelings of not being able to take effective action to significantly mitigate climate change risks. ‘Solastalgia’ refers to “the distress and isolation caused by the gradual removal of solace from the present state of one’s home environment” [29]. The term ‘solastalgia’ is also commonly referred to throughout much of the literature on climate change and mental health to articulate the feelings associated with displacement following a climate change-related extreme weather event [17]. This new vocabulary provides the language to explore some of the broader mental health implications of escalating climate change risks.

For many people, climate change is experienced by way of vicarious threats or as an existential threat to civilisation [37]. People may experience vicarious threats when they receive weather warnings related to future disaster seasons or when they hear about environmental stressors experienced by people in other places. For many people, this is largely how climate change is experienced—not as a direct threat, but as a global threat, often distant in time and place, or as a threat to our very way of life. Qualitative research finds evidence of some people being deeply affected by feelings of loss, helplessness, and frustration as they engage with the problems of global climate change [97].

### Part 3: Priority actions to address climate change and mental health

Acting on the health consequences of climate change requires actions rooted in both mitigation and adaptation at all levels—from global to local—and from all sectors and individuals. Climate change mitigation refers to overarching efforts to reduce greenhouse gas emissions and enhance carbon sinks to slow the speed, scale, and magnitude of climate change [98]. Key climate change mitigation priorities include reducing energy demand (through reduced consumption and increased energy efficiency); a swift and equitable transition from fossil fuels to renewable energy; reducing emissions from agriculture and forestry; and strengthening land-based emissions sequestration. Climate change adaptation refers to interventions that respond to the effects of climate change by adjusting, moderating, and coping with the risks and impacts of climate change [98]. Adaptation is ultimately affected by the *capacity* to adapt, which is the ability and willingness to respond to climate change mediated by individual and collective agency [99]. Adaptive capacity is determined by things like: governance, economics, infrastructure, technology, information and skills, institutions, and equity [100]. Examples of adaptation interventions that address climate change and health include: surveillance and monitoring of disease burdens related to climate change and health; education (e.g. public health promotion of the risks of vector-borne illness), and capacity building (e.g. psychological first-aid, and surge capacities at hospitals and health care facilities); preparing for extreme weather events; and re-locating entire communities to geographic areas where sea-level rise and frequent extreme weather events are less-likely to occur [7].

Within international approaches to combat climate change there is a significant focus not only on mitigation but also adaptation. From the Paris Accord to the Lancet Countdown on Climate Change, to the Planetary Manifesto and climate action marches—policy makers, academics, and the general population are taking steps to mitigate and adapt to the current threats and impacts to preserve a future for the next generation [4, 39]. These actions, however, often fail to address the gap between stated goals of emissions reduction commitments and the speed of actions required to keep global warming well below 1.5–2 °C [101].

With a specific focus on mental health and climate change, there are a number of global programs in place that indirectly address the topic area—like for example measures to enhance and protect mental health in the Sustainable Development Goals 2016–2030 [102]; efforts by the Movement for Global Mental Health to increase the holistic conceptualization of health to incorporate mental health [103]; the Sendai Framework, a 15-year disaster risk reduction program [104]; and, the United Nations Human Settlement Program that promotes sustainable urban development [105]. There is a need, however, to harness health and mental health related synergies amongst these global agreements since none of these in and of themselves will likely be sufficient to address the future risks and impacts of climate change.

Coordinated, collaborative efforts to address the mental health implications of climate change not only require policy frameworks but also concrete actions on behalf of mental health practitioners. Such concrete actions may include: communicating about climate change and mental health in a way that helps people to see that it is relevant and salient to them; advocacy for greenhouse gas reductions in health care facilities and engagement in efforts to reduce the environmental footprint of the health care sector; and, engaging in adaptation measures like preparing for and responding to extreme events.

#### Psychological adaptation

Psychological adaptation requires a set of responses, it requires an acknowledgement of the grave threats posed by climate change and the profoundly consequential global crisis. It requires coping strategies to manage the feelings and thoughts that arise so that people can face up to, and come to terms with, these threats and consequences rather than avoiding the creeping problem of climate change. It also requires behavioural and psychological engagement, in which people change and adjust their behaviour and lifestyle in order to reduce the threat and protect themselves.

Active hope—something Macy and Johnstone champion—supports psychological adaptation. Active hope is required to move hopeful intentions from a passive state where waiting for someone else to take-on the task of addressing the climate change problem is replaced with an active process of climate change mitigation and adaptation behaviours [106]. The key point here is that hope alone cannot provide sufficient protection from the escalating risks of climate change. This active process occurs when the reality of the problem is acknowledged as is the magnitude of the problem, intentions to address the problem are set, and engaged actions take place. While these three steps may oversimplify the complexity of acting in the face of bureaucracy, climate denialism, or downright avoidance and ignorance of the magnitude of the problem area, these three steps are indeed the pivot points of transformation. These pivot points, however, need to be upheld by global political will and policy commitments that tackle the problem at the appropriate scale and speed. To do so, public awareness of the severity, magnitude and range of health impacts—current and projected—is required to pressure governments and communities to act now. Also, discernible interventions are needed to demonstrate a tangible path forward to respond to the risks and impacts we face in a changing climate. Examples of these types of interventions are explored below.

#### Adaptation measures

Adaptation measures that address the psychosocial impacts of climate change come in a variety of forms, i.e. policies, practices, behavioral interventions, community-based interventions, specific training, and pharmacotherapeutics. Some general approaches to address climate change-related mental health problems or illnesses include: primary care interventions, individual and group-based therapy, cognitive based interventions (including cognitive based therapy, cognitive restructuring, and, stress inoculation training), and crisis counselling [67]. More broadly, emotional resiliency may be sustained by engaging with art, literature, and spirituality. In addition to the above, the list below contains some specific priority adaptation mechanisms that ought to be considered to support population-level mental health in a changing climate:

- Policy responses: improving access and funding to mental health care;
- Surveillance and monitoring: administering epidemiological surveys after extreme weather events, and monitoring emergency department visits during heat waves and following extreme weather events;
- Practice: the application of a stepped-care approach to mental health that is often used in disaster mental health to support different levels of interventions depending on the timing of the disaster and the level of distress (see [107, 108]);
- Preparation and response: climate change adaptation/resilience planning in the mental health system;
- Community-based interventions: climate change resilience plans that address psychosocial wellbeing; and,
- Special training for care providers and first responders: e.g. psychological first aid.

Other innovative approaches to addressing mental health and wellbeing in a changing climate writ large include experiencing and preserving nature. Koger et al. suggest that environmental preservation provides people with a sense of stewardship and personal investment that can help people overcome feelings of hopelessness, anxiety, and ecoparalysis [109]. Koger et al. suggest: “if people feel a deep connection to places, wilderness, and other species, then threats to these others are much more likely to be viewed as personal issues” [109]. Other research on the restorative benefits of natural environments and settings has found that biodiversity in natural environments is important for human health and wellbeing and has a particularly positive effect on mood, attention and cognition [110]. A common practice in Japan to reduce stress and anxiety is the practice of shinrin-yoku, otherwise referred to as forest bathing. In a study by Lee et al., authors found that forest bathing resulted in decreased cortisol levels, pulse rates, and negative feelings and significantly increased positive feelings [111]. Research on people’s interactions with nationally important ecosystems, like World Heritage Areas for example, highlights positive impacts including quality of life, a sense of place and belonging, self-identity, restoration and inspiration [112].

While there are a number of interventions to support psychosocial wellbeing within a changing climate, it is important to highlight that many of these interventions are still quite nascent and administered in ad hoc fashion, and these interventions are mainly accessible in developed countries. Sustainable mental health care in developed and developing nations is urgently needed as the realities of climate change become more and more apparent—especially for those most marginalized. Further, there are research needs in this domain where the efficacy and accessibility of mental health interventions-related to climate change are assessed.

## Conclusion

Climate change affects mental health in a variety of direct, indirect, and overarching pathways—disproportionately affecting those most marginalized. The mental health implications of climate change can result in mental problems and illness as well as affirmative psychosocial outcomes. While the timing and triggers associated with climate change and mental health may vary, making it challenging to establish the manifold links between climate change and mental health, the opportunities of attributing mental health to climate change support climate mitigation as well as mental health action and psychosocial resiliency. Global commitments, like the Paris Accord, the SDGs, and the Sendai Framework are needed to help advance global mental health and climate action; however, coordination amongst these commitments is required—as are concrete actions on behalf of health practitioners—if the issue of mental health and a changing climate is to be efficiently and holistically addressed. Further, a reckoning with social, environmental, and climate injustice is needed if actions to address climate change and mental health are to be rooted in health equity. Transformative action—where inequities are addressed, active hope is demonstrated, and communities are mobilized—is the defining opportunity of the twenty-first century to address the climate change impacts on mental health.

## Footnotes

1 This article builds on an earlier overview of mental health and climate change by Fritze et al. [37].

## References

[1] World Health Organization. WHO calls for urgent action to protect health from climate change—sign the call. 2015. http://www.who.int/globalchange/global-campaign/cop21/en/. Accessed 11 Dec 2017.

[2] Intergovernmental Panel on Climate Change. Summary for policymakers. In: Field, CB, Barros V, Stocker TF, Qin D, Dokken DJ, Ebi KL, Mastrandrea MD, Mach KJ, Plattner G-K, Allen SK, Tignor M, and Midgley PM, editors. Managing the risks of extreme events and disasters to advance climate change adaptation. A special report of working groups I and II of the intergovernmental panel on climate change. New York: Cambridge University Press; 2012.

[3] Costello A, Abbas M, Allen A, Ball S, Bell S, Bellamy R, Lee M (2009). Managing the health effects of climate change. Lancet.

[4] Watts N, Amann M, Ayeb-Karlsson S, Belesova K, Bouley T, Boykoff M, Byass P, Cai W, Campbell-Lendrum D, Chambers J, Cox PM, Daly M, Dasandi N, Davies M, Depledge M, Depoux A, Dominguez-Salas P, Drummond P, Ekins P, Flahault A, Frumkin H, Georgeson L, Ghanei M, Grace D, Graham H, Grojsman R, Haines A, Hamilton I, Hartinger S, Johnson A, Kelman I, Kiesewetter G, Kniveton D, Liang L, Lott M, Lowe R, Mace G, Odhiambo Sewe M, Maslin M, Mikhaylov S, Milner J, Latifi AM, Moradi-Lakeh M, Morrissey K, Murray K, Neville T, Nilsson M, Oreszczyn T, Owfi F, Pencheon D, Pye S, Rabbaniha M, Robinson E, Rocklöv J, Schütte S, Shumake-Guillemot J, Steinbach R, Tabatabaei M, Wheeler N, Wilkinson P, Gong P, Montgomery H, Costello A (2017). The Lancet countdown on health and climate change: from 25 years of inaction to a global transformation for public health. Lancet.

[5] Watts N, Adger WN, Agnolucci P, Blackstock J, Byass P, Cai W, Chaytor S, Colbourn T, Collins M, Cooper A, Cox PM, Depledge J, Drummond P, Ekins P, Galaz V, Grace D, Graham H, Grubb M, Haines A, Hamilton I, Hunter A, Jiang X, Li M, Kelman I, Liang L, Lott M, Lowe R, Luo Y, Mace G, Maslin M, Nilsson M, Oreszczyn T, Pye S, Quinn T, Svensdotter M, Venevsky S, Warner K, Xu B, Yang J, Yin Y, Yu C, Zhang Q, Gong P, Montgomery H, Costello A (2015). Health and climate change: policy responses to protect public health. Lancet..

[6] United States Government Accountability Office. Climate change: information on potential economic effects could help guide federal efforts to reduce fiscal exposure. 2017. http://www.gao.gov/assets/690/687466.pdf. Accessed 12 Nov 2017.

[7] Berry P, Clarke K-L, Parker S, Warren FJ, Lemmen DS (2014). Chapter 7: human health. Canada in a changing climate: sector perspectives on impacts and adaptation.

[8] Friedli L. Mental health, resilience and inequalities. World Health Organization. 2009. http://www.euro.who.int/__data/assets/pdf_file/0012/100821/E92227.pdf. Accessed 12 Dec 2017.

[9] Butler CD, Bowles DC, McIver L, Page L (2014). Mental health, cognition and the challenge of climate change. Clim Change Glob Health.

[10] CAMH. Mental illness and addictions: facts and statistics. 2012. http://www.camh.ca/en/hospital/about_camh/newsroom/for_reporters/Pages/addictionmentalhealthstatistics.aspx. Accessed 6 Jan 2015.

[11] World Health Organization. Mental health. 2017. http://www.who.int/mental_health/en/. Accessed 10 Nov 2017.

[12] McMichael AJ, Woodruff RE, Hales S (2006). Climate change and human health: present and future risks. Lancet.

[13] Berry HL, Kelly BJ, Hanigan IC, Coates JH, McMichael AJ, Welsh JA, Kjellstrom T (2008). Rural mental health impacts of climate change. Commissioned report for the Garnaut Climate Change Review.

[14] Berry H (2009). Pearl in the oyster: climate change as a mental health opportunity. Aust Psychiatry..

[15] Berry HL, Bowen K, Kjellstrom T (2010). Climate change and mental health: a causal pathways framework. Int J Public Health..

[16] Bourque F, Cunsolo Willox A (2014). Climate change: the next challenge for public mental health?. Int Rev Psychiatry..

[17] Willox AC, Harper SL, Ford JD, Landman K, Houle K, Edge VL (2012). “From this place and of this place:” climate change, sense of place, and health in Nunatsiavut, Canada. Soc Sci Med.

[18] Willox AC, Harper SL, Edge VL, Landman K, Houle K, Ford JD (2013). The land enriches the soul: on climatic and environmental change, affect, and emotional health and well-being in Rigolet, Nunatsiavut, Canada. Emotion Space Soc.

[19] Willox AC, Harper SL, Ford JD, Edge VL, Landman K, Houle K, Blake S, Wolfrey C (2013). Climate change and mental health: an exploratory case study from Rigolet, Nunatsiavut, Canada. Clim Change..

[20] Willox AC, Stephenson E, Allen J, Bourque F, Drossos A, Elgarøy S, Kral MJ, Mauro I, Moses J, Pearce T, MacDonald JP (2015). Examining relationships between climate change and mental health in the Circumpolar North. Reg Environ Change.

[21] Doherty TJ, Clayton S (2011). The psychological impacts of global climate change. Am Psychol.

[22] Clayton S, Manning C, Hodge C (2014). Beyond storms & droughts: the psychological impacts of climate change.

[23] Clayton S, Manning C, Krygsman K, Speiser M (2017). Mental health and our changing climate: impacts, implications, and guidance.

[24] Coyle KJ, Van Susteren L. The psychological effects of global warming on the United States: and why the US mental health care system is not adequately prepared. National Wildlife Federation. 2012. http://www.climateaccess.org/sites/default/files/NWF_Psychological%20Effects.pdf. Accessed 12 Nov 2017.

[25] Weissbecker I (2011). Climate change and human well-being: global challenges and opportunities.

[26] Swim J, Clayton S, Doherty T, Gifford R, Howard G, Reser J, Stern P, Weber E (2009). Psychology and global climate change: addressing a multi-faceted phenomenon and set of challenges. A report by the American Psychological Association’s task force on the interface between psychology and global climate change.

[27] Nurse J, Basher D, Bone A, Bird W (2010). An ecological approach to promoting population mental health and well-being—a response to the challenge of climate change. Perspect Public Health.

[28] Agnew R (2012). Dire forecast: a theoretical model of the impact of climate change on crime. Theor Criminol.

[29] Albrecht G. Chronic environmental change: emerging ‘psychoterratic’syndromes. In: Climate change and human well-being. New York: Springer; 2011. p. 43–56.

[30] Ramsay T, Manderson L, Weissbecker (2011). Resilience, spirituality and posttraumatic growth: reshaping the effects of climate change. Climate change and human well-being.

[31] Edwards T, Wiseman J, Weissbecker (2011). Climate change, resilience and transformation: challenges and opportunities for local communities. Climate change and human well-being.

[32] McMichael A (2017). climate change and the health of nations: famines, fevers, and the fate of populations.

[33] Trombley J, Chalupka S, Anderko L (2017). Climate change and mental health. AJN Am J Nurs.

[34] Gamble JL, Balbus J, Berger M, Bouye K, Campbell V, Chief K, Conlon K, Crimmins A, Flanagan B, Gonzalez-Maddux C, Hallisey ECh (2016). 9: populations of concern.

[35] Cusack L, de Crespigny C, Athanasos P (2011). Heatwaves and their impact on people with alcohol, drug and mental health conditions: a discussion paper on clinical practice considerations. J Adv Nurs.

[36] Page LA, Hajat S, Kovats RS, Howard LM (2012). Temperature-related deaths in people with psychosis, dementia and substance misuse. Br J Psychiatry..

[37] Fritze JG, Blashki GA, Burke S, Wiseman J (2008). Hope, despair and transformation: climate change and the promotion of mental health and wellbeing. Int J Ment Health Syst.

[38] Hancock T, Spady DW, Soskolne CL, editors. Global change and public health: addressing the ecological determinants of health—the report in brief. 2015. http://www.cpha.ca/uploads/policy/edh-brief.pdf. Accessed 7 Nov 2017.

[39] Horton R, Beaglehole R, Bonita R, Raeburn J, McKee M, Wall S (2014). From public to planetary health: a manifesto. Lancet..

[40] Murray CJ, Vos T, Lozano R, Naghavi M, Flaxman AD, Michaud C, Ezzati M, Shibuya K, Salomon JA, Abdalla S, Aboyans V (2013). Disability-adjusted life years (DALYs) for 291 diseases and injuries in 21 regions, 1990–2010: a systematic analysis for the global burden of disease study 2010. Lancet..

[41] Becker AE, Kleinman A (2013). Mental health and the global agenda. N Engl J Med.

[42] Vigo D, Thornicroft G, Atun R (2016). Estimating the true global burden of mental illness. Lancet Psychiatry..

[43] Patel V, Saxena S, Frankish H, Boyce N (2016). Sustainable development and global mental health—a Lancet Commission. Lancet..

[44] Links J. Predicting community resilience and recovery after a disaster. CDC. 2017. https://blogs.cdc.gov/publichealthmatters/2017/08/predicting-community-resilience-and-recovery-after-a-disaster/. Accessed 1 Sept 2017.

[45] National Academies of Sciences (2016). Engineering, and medicine. Attribution of extreme weather events in the context of climate change.

[46] Stott PA, Stone DA, Allen MR (2004). Human contribution to the European heatwave of 2003. Nature.

[47] Stone DA, Allen MR (2005). The end-to-end attribution problem: from emissions to impacts. Clim Change..

[48] Easterling DR, Kunkel KE, Wehner MF, Sun L (2016). Detection and attribution of climate extremes in the observed record. Weather Clim Extremes..

[49] Pall P, Aina T, Stone DA, Stott PA, Nozawa T, Hilberts AG, Lohmann D, Allen MR (2011). Anthropogenic greenhouse gas contribution to flood risk in England and Wales in autumn 2000. Nature.

[50] Otto FEL, Massey N, van Oldenborgh GJ, Jones RG, Allen MR (2012). Reconciling two approaches to attribution of the 2010 Russian heat wave. Geophys Res Lett..

[51] Tunstall S, Tapsell S, Green C, Floyd P, George C (2006). The health effects of flooding: social research results from England and Wales. J Water Health..

[52] Waite TD, Chaintarli K, Beck CR, Bone A, Amlôt R, Kovats S, Reacher M, Armstrong B, Leonardi G, Rubin GJ, Oliver I (2017). The English national cohort study of flooding and health: cross-sectional analysis of mental health outcomes at year one. BMC Public Health..

[53] Alderman K, Turner LR, Tong S (2013). Assessment of the health impacts of the 2011 summer floods in Brisbane. Disaster Med Public Health Preparedness..

[54] Fernandez A, Black J, Jones M, Wilson L, Salvador-Carulla L, Astell-Burt T, Black D (2015). Flooding and mental health: a systematic mapping review. PLoS One..

[55] Stanke C, Murray V, Amlôt R, Nurse J, Williams R (2012). The effects of flooding on mental health: outcomes and recommendations from a review of the literature. PLoS Curr..

[56] Chakrabhand S, Panyayong B, Sirivech P (2006). Mental health and psychosocial support after the tsunami in Thailand. Int Rev Psychiatry..

[57] Norris FH, Friedman MJ, Watson PJ, Byrne CM, Diaz E, Kaniasty K (2002). 60,000 disaster victims speak: part I. An empirical review of the empirical literature, 1981–2001. Psychiatry..

[58] Vins H, Bell J, Saha S, Hess JJ (2015). The mental health outcomes of drought: a systematic review and causal process diagram. Int J Environ Res Public Health..

[59] Sahni V, Scott AN, Beliveau M, Varughese M, Dover DC, Talbot J (2016). Public health surveillance response following the southern Alberta floods, 2013. Can J Public Health.

[60] Whaley AL (2009). Trauma among survivors of Hurricane Katrina: considerations and recommendations for mental health care. J Loss Trauma..

[61] North CS, Pfefferbaum B (2013). Mental health response to community disasters: a systematic review. JAMA.

[62] Azuma K, Ikeda K, Kagi N, Yanagi U, Hasegawa K, Osawa H (2014). Effects of water-damaged homes after flooding: health status of the residents and the environmental risk factors. Int J Environ Health Res.

[63] Crabtree A (2012). Climate change and mental health following flood disasters in developing countries, a review of the epidemiological literature: what do we know, what is being recommended?. Aust J Disaster Trauma Stud.

[64] Eisenman D, McCaffrey S, Donatello I, Marshal G (2015). An ecosystems and vulnerable populations perspective on solastalgia and psychological distress after a wildfire. EcoHealth.

[65] Kessler RC, Galea S, Gruber MJ, Sampson NA, Ursano RJ, Wessely S (2008). Trends in mental illness and suicidality after Hurricane Katrina. Mol Psychiatry..

[66] Greene G, Paranjothy S, Palmer SR (2015). Resilience and vulnerability to the psychological harm from flooding: the role of social cohesion. Am J Public Health.

[67] Anderson H, Brown C, Cameron LL, Christenson M, Conlon KC, Dorevitch S. Climate and health intervention assessment: evidence on public health interventions to prevent the negative health effects of climate change. Climate and health technical report series. BRACE Midwest and Southeast Community of Practice. Climate and Health Program, Centers for Disease Control and Prevention. 2017.

[68] Vestal C. ‘Katrina brain’: the invisible long-term toll of megastorms. POLITICO. 2017. http://www.politico.com/agenda/story/2017/10/12/psychological-toll-natural-disasters-000547. Accessed 12 Oct 2017.

[69] World Health Organization. Operational framework for building climate resilient health systems. 2015. http://www.who.int/globalchange/publications/building-climate-resilient-health-systems/en/. Accessed 20 Sept 2017.

[70] Giddens A. The politics of climate change. UK: Cambridge; 2009.

[71] Marshall G (2015). Don’t even think about it: why our brains are wired to ignore climate change.

[72] Chand PK, Murthy P (2008). Climate change and mental health. Reg Health Forum..

[73] Wang H, Horton R (2015). Tackling climate change: the greatest opportunity for global health. Lancet..

[74] Dodgen D, Donato D, Kelly N, La Greca A, Morganstein J, Reser J, Ruzek J, Schweitzer S, Shimamoto MM, Tart KT, Ursano RCh (2016). 8: mental health and well-being.

[75] Bryant R, Waters E, Gibbs L, Gallagher C, Pattison P, Lusher D, MacDougall C, Harms L, Block K, Snowdon E, Sinnott V, Ireton G, Richardson J, Forbes D (1014). Psychological outcomes following the Victorian Black Saturday bushfires. Aust NZ J Psychiatry.

[76] Burton H, Rabito F, Danielson L, Takaro TK (2016). Health effects of flooding in Canada: a 2015 review and description of gaps in research. Can Water Resourc J/Revue canadienne des ressources hydriques..

[77] Neria Y, Shultz JM (2012). Mental health effects of Hurricane Sandy: characteristics, potential aftermath, and response. JAMA.

[78] Schmeltz MT, González SK, Fuentes L, Kwan A, Ortega-Williams A, Cowan LP (2013). Lessons from hurricane sandy: a community response in Brooklyn, New York. J Urban Health..

[79] Osofsky JD, Osofsky HJ, Kronenberg M, Hansel TC. The aftermath of Hurricane Katrina: mental health considerations and lessons learned, chapter 10. In: Helping families and communities recover from disaster. Washington, D.C: American Psychological Association; 2010. p. 241–63.

[80] Haskett ME, Scott SS, Nears K, Grimmett MA (2008). Lessons from Katrina: disaster mental health service in the Gulf Coast region. Prof Psychol.

[81] Galea S, Brewin CR, Gruber M, Jones RT, King DW, King LA, McNally RJ, Ursano RJ, Petukhova M, Kessler RC (2007). Exposure to hurricane-related stressors and mental illness after Hurricane Katrina. Arch Gen Psychiatry.

[82] Rhodes J, Chan C, Paxson C, Rouse CE, Waters M, Fussell E (2010). The impact of hurricane Katrina on the mental and physical health of low-income parents in New Orleans. Am J Orthopsychiatry.

[83] Preti A, Miotto P (1998). Seasonality in suicides: the influence of suicide method, gender and age on suicide distribution in Italy. Psychiatry Res..

[84] Reifels L, Spittal MJ, Dückers M, Mills KL, Pirkis J (2018). Suicidality risk and (repeat) disaster exposure: findings from a nationally representative population survey. Psychiatry Interpers Biolog Process..

[85] Solnit R (2010). A paradise built in hell: the extraordinary communities that arise in disaster.

[86] OBrien LV, Berry HL, Coleman C, Hanigan IC (2014). Drought as a mental health exposure. Environ Res..

[87] Yusa A, Berry P, Cheng J, Ogden N, Bonsal B, Stewart R, Waldick R (2015). Climate change, drought and human health in Canada. Int J Environ Res Public Health..

[88] Stain HJ, Kelly B, Lewin TJ, Higginbotham N, Beard JR, Hourihan F (2008). Social networks and mental health among a farming population. Soc Psychiatry Psychiatr Epidemiol.

[89] Ellis NR, Albrecht GA (2017). Climate change threats to family farmers’ sense of place and mental wellbeing: a case study from the Western Australian wheatbelt. Soc Sci Med.

[90] Gleick PH (2014). Water, drought, climate change, and conflict in Syria. Weather Clim Soc.

[91] IEHS. 5 Facts on climate migrants. United Nations University. 2015. https://ehs.unu.edu/blog/5-facts/5-facts-on-climate-migrants.html. Accessed 12 Dec 2017.

[92] Department of Defense. National security implications of climate-related risks and a changing climate. 2015. http://archive.defense.gov/pubs/150724-congressional-report-on-national-implications-of-climate-change.pdf?source=govdelivery. Accessed 14 Dec 2017.

[93] Gemenne F, Barnett J, Adger WN, Dabelko GD (2014). Climate and security: evidence, emerging risks, and a new agenda. Clim Change..

[94] Aljazeera. UN: number of Syrian refugees passes five million. 2017. http://www.aljazeera.com/news/2017/03/number-syrian-refugees-passes-million-170330132040023.html. Accessed 14 Dec 2017.

[95] Siriwardhana C, Stewart R (2013). Forced migration and mental health: prolonged internal displacement, return migration and resilience. Int Health..

[96] Albrecht G, Sartore GM, Connor L, Higginbotham N, Freeman S, Kelly B, Stain H, Tonna A, Pollard G (2007). Solastalgia: the distress caused by environmental change. Aust Psychiatry..

[97] Moser SC (2013). Navigating the political and emotional terrain of adaptation: community engagement when climate change comes home. Successful adaptation to climate change: linking science and policy in a rapidly changing world.

[98] Allwood JM, Bosetti V, Dubash NK, Gómez-Echeverri L, von Stechow C, Edenhofer O, Pichs-Madruga R, Sokona Y, Farahani E, Kadner S, Seyboth K, Adler A, Baum I, Brunner S, Eickemeier P, Kriemann B, Savolainen J, Schlömer S, von Stechow C, Zwickel T, Minx JC (2014). Glossary. Climate change 2014: mitigation of climate change Contribution of working group III to the fifth assessment report of the Intergovernmental Panel on Climate Change.

[99] Brown K, Westaway E (2011). Agency, capacity, and resilience to environmental change: lessons from human development, well-being, and disasters. Ann Rev Environ Resourc..

[100] Séguin J, Berry P, Bouchet V, Clarke KL, Furgal C, Environmental I, MacIver D. Human health in a changing climate: a Canadian assessment of vulnerabilities and adaptive capacity. In: Human health in a changing climate. 2008.

[101] UN Environment. Emissions gap report. UNEP Synthesis Report. 2017. https://www.unenvironment.org/resources/emissions-gap-report. Accessed 23 Feb 2018.

[102] United Nations. Sustainable development goals. 2017. https://sustainabledevelopment.un.org/sdgs. Accessed 2 Jan 2018.

[103] Movement for Global Mental Health. N.d. http://www.globalmentalhealth.org. Accessed 2 Jan 2018.

[104] United Nations Office for Disaster Risk Reduction. Sendai framework for disaster risk reduction. 2015. http://www.unisdr.org/we/coordinate/sendai-framework. Accessed 2 Jan 2018.

[105] United Nations. United Nations human settlements programme (UN-HABITAT). 2015. https://www.un-ngls.org/index.php/engage-with-the-un/un-civil-society-contact-points/144-united-nations-human-settlements-programme-un-habitat. Accessed 2 Jan 2018.

[106] Macy J, Johnstone C (2012). Active hope: how to face the mess we’re in without going crazy.

[107] Bower P, Gilbody S (2005). Stepped care in psychological therapies: access, effectiveness and efficiency. Br J Psychiatry..

[108] Twomey C, Byrne M (2012). A stepped care approach to mental health. Forum..

[109] Koger SM, Leslie KE, Hayes ED (2011). Climate change: psychological solutions and strategies for change. Ecopsychology..

[110] Maas J, Verheij R, Groenewegen P, de Vries S, Spreeuwenberg P (2006). Green space, urbanity and health: how strong is the relationship?. J Epidemiol Community Health.

[111] Lee J, Park BJ, Tsunetsugu Y, Ohira T, Kagawa T, Miyazaki Y (2011). Effect of forest bathing on physiological and psychological responses in young Japanese male subjects. Public Health..

[112] Bentrupperbäumer JM, Reser JP, Stork N, Turton S (2008). Encountering a World Heritage landscape: community and visitor perspectives and experiences. Living in a dynamic tropical forest landscape: lessons from Australia.

